# Oral Contraceptive-Induced Cerebral Venous Thrombosis: A Case Report

**DOI:** 10.7759/cureus.78407

**Published:** 2025-02-03

**Authors:** Shami F Amri, Najlaa M Alsudairy

**Affiliations:** 1 College of Pharmacy, Jazan University, Jazan, SAU; 2 Family Medicine, National Guard Hospital, Jeddah, SAU

**Keywords:** anticoagulation, cerebral venous thrombosis, estrogen-induced thrombosis, hypercoagulability, magnetic resonance venography, oral contraceptive pills, prothrombotic state, stroke in young adults, thromboembolism, venous infarction

## Abstract

Cerebral venous thrombosis (CVT) is a rare, potentially life-threatening condition that occurs when blood clots form in the venous sinuses of the brain, leading to impaired venous drainage, increased intracranial pressure, and neurological deficits. We discuss the case of a 32-year-old female who presented with a four-day history of worsening headache, nausea, vomiting, and right-sided weakness. She was on oral contraceptive pills (OCPs) for two years for menstrual regulation. Neuroimaging, including non-contrast CT, CT venography, and MRI with MR venography, confirmed the diagnosis of CVT involving the superior sagittal sinus and right transverse sinus, with evidence of venous infarction. Laboratory investigations showed elevated D-dimer, but thrombophilia testing was negative. The patient was immediately started on anticoagulation therapy with low-molecular-weight heparin (LMWH), later transitioned to warfarin, and received supportive care. Symptoms improved significantly within days, and follow-up imaging at three months showed partial recanalization. The patient was advised to discontinue OCPs permanently and provided counseling on alternative contraceptive methods. This report emphasizes the importance of early recognition, timely neuroimaging, and appropriate anticoagulation in the management of OCP-associated CVT. Long-term follow-up is crucial to monitor for recanalization and to reduce the risk of recurrence.

## Introduction

Cerebral venous thrombosis (CVT) is a rare but potentially life-threatening cause of stroke, accounting for approximately 0.5-1% of all strokes. It occurs due to thrombosis of the cerebral venous sinuses or cortical veins, leading to impaired venous drainage, increased intracranial pressure, and, in severe cases, venous infarction or hemorrhage [1,2]. Unlike arterial strokes, CVT predominantly affects young adults, with a marked female predominance due to hormonal influences. Risk factors include pregnancy, the postpartum period, thrombophilic disorders, malignancy, infections, and the use of exogenous hormones such as oral contraceptive pills (OCPs) [1-3].

OCPs are widely used for contraception and menstrual regulation, but their association with an increased risk of venous thromboembolism, including CVT, has been well documented. Estrogen-containing contraceptives contribute to a prothrombotic state by increasing coagulation factors, reducing anticoagulant proteins, and impairing fibrinolysis [2-4]. Despite this known risk, CVT remains underrecognized, often leading to delayed diagnosis and treatment. Clinical presentation is highly variable, ranging from isolated headaches to focal neurological deficits, seizures, or coma, necessitating a high index of suspicion [3,5]. This report highlights the diagnostic and management challenges of OCP-associated CVT and underscores the importance of early recognition, prompt anticoagulation, and long-term follow-up to optimize patient outcomes.

## Case presentation

A previously healthy 32-year-old female presented to the emergency department with a four-day history of persistent headache, which had progressively worsened in intensity, and was unresponsive to over-the-counter analgesics. The headache was holocranial, with a pressure-like quality, and associated with nausea, intermittent vomiting, and transient episodes of blurred vision. She denied photophobia, phonophobia, or recent febrile illness. A day before admission, she had experienced new-onset right-sided weakness and paresthesia, accompanied by mild dysarthria. There was no history of seizures, head trauma, recent surgery, prolonged immobilization, or known hypercoagulable disorder. She had no personal or family history of thromboembolic events. Her medical history was unremarkable except for the use of combined OCPs for the past two years for menstrual regulation. She was a non-smoker, had no history of illicit drug use, and denied recent long-haul travel.

On arrival, the patient was alert and oriented, with a Glasgow Coma Scale (GCS) score of 15. Her vital signs were within normal limits, with a blood pressure of 118/76 mmHg, heart rate of 82 beats per minute, respiratory rate of 16 breaths per minute, and oxygen saturation of 98% on room air. Neurological examination revealed mild right-sided hemiparesis (Medical Research Council grade 4/5), hypoesthesia over the right upper and lower extremities, and mild dysarthria. Cranial nerves were intact, deep tendon reflexes were slightly brisk on the right side, and plantar response was equivocal bilaterally. Fundoscopic examination showed mild bilateral papilledema. Cardiovascular, respiratory, and abdominal examinations were unremarkable.

Initial laboratory investigations, including complete blood count, renal and liver function tests, coagulation profile, and inflammatory markers, were within normal limits. D-dimer was mildly elevated (780 ng/mL; reference range: <500 ng/mL). A thrombophilia workup, including protein C and S levels, antithrombin III, lupus anticoagulant, anticardiolipin antibodies, and factor V Leiden mutation, was sent for evaluation.

**Figure 1:**
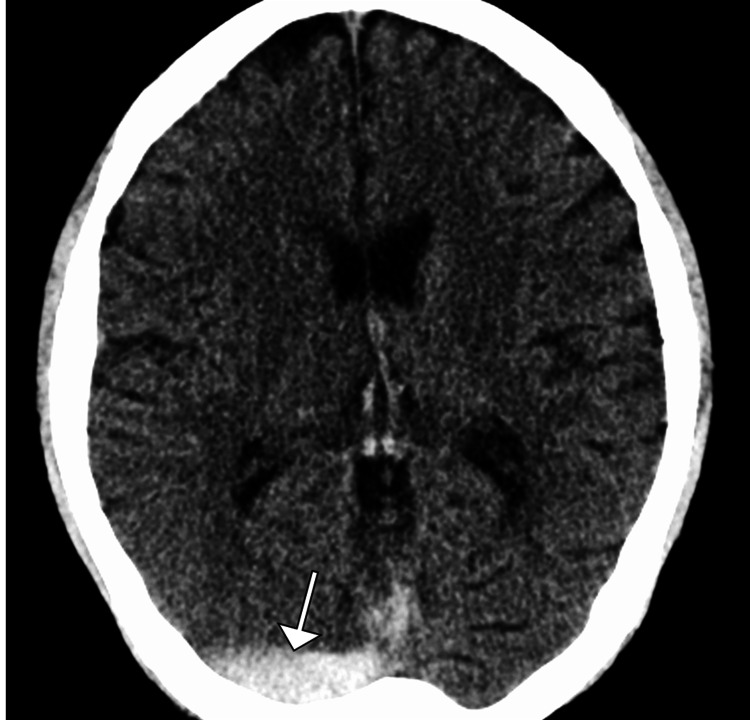
Axial non-contrast CT head shows hyperdensity along the right straight and transverse sinus (arrow) CT: computed tomography

**Figure 2:**
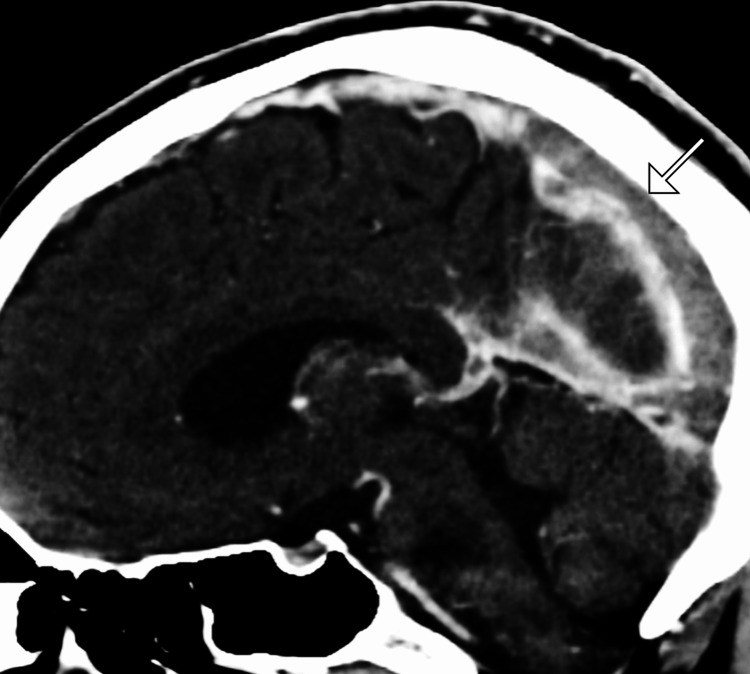
Sagittal CTV shows filling defect along the superior sagittal sinus (arrow) CTV: computed tomography venography

**Figure 3:**
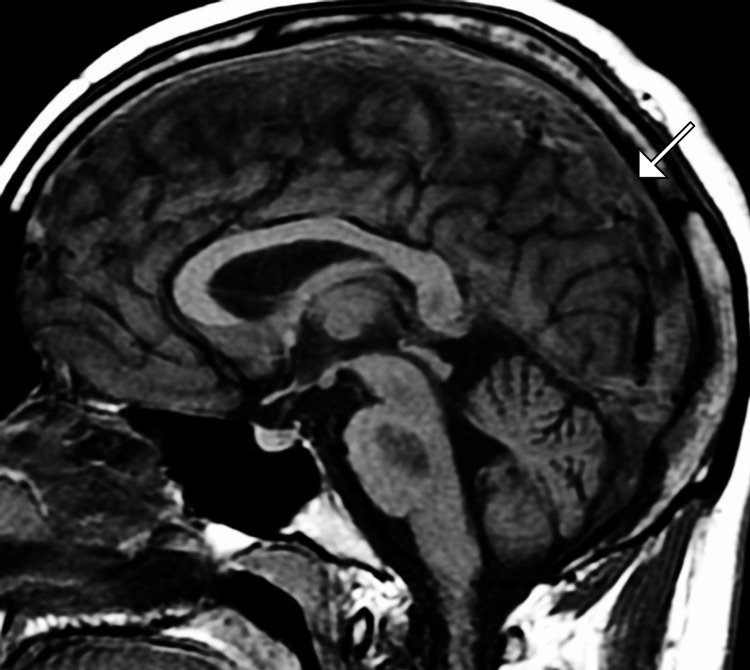
Sagittal MRI image shows an acute thrombus in the superior sagittal sinus (arrow) MRI: magnetic resonance imaging

Non-contrast CT of the brain revealed subtle hyperdensity along the superior sagittal sinus, suggestive of a possible thrombus. To further characterize the findings, a CT venography (CTV) was performed, confirming extensive thrombosis of the superior sagittal sinus and right transverse sinus with associated mild venous congestion. Magnetic resonance imaging (MRI) of the brain with MR venography (MRV) showed restricted diffusion in the left frontoparietal region, indicating a venous infarct secondary to CVT. No evidence of hemorrhagic transformation was noted (Figures 1-3).

Given the history of the patient's OCP use, elevated D-dimer, and radiological confirmation of CVT, a diagnosis of OCP-associated cerebral venous thrombosis was established. The patient was immediately started on therapeutic anticoagulation with low-molecular-weight heparin (LMWH) at a therapeutic dose, later transitioning to an oral vitamin K antagonist (warfarin) with INR monitoring. Supportive management included hydration, analgesia for headache control, and acetazolamide for raised intracranial pressure due to papilledema.

During hospitalization, her neurological symptoms gradually improved. By day five, right-sided weakness had resolved significantly, with near-complete motor recovery. Repeat fundoscopic examination showed resolving papilledema. She was monitored closely for any signs of clinical deterioration or hemorrhagic complications. A thrombophilia workup later returned negative, reinforcing the diagnosis of OCP-induced CVT as the primary etiology.

The patient was discharged on warfarin with a target INR of 2-3 and advised to discontinue OCPs permanently. She was counseled on alternative non-hormonal contraceptive options. A structured follow-up plan was established, with neurology and hematology outpatient visits scheduled at two weeks, one month, and three months. A repeat MRV at three months demonstrated partial recanalization of the superior sagittal and right transverse sinuses. At six months, the patient remained asymptomatic, with complete neurological recovery, and warfarin was discontinued under hematologic guidance. She was advised on lifestyle modifications and the potential risks of future thrombotic events, especially in the context of hormonal therapy.

## Discussion

CVT is a rare but serious cerebrovascular disorder that differs significantly from arterial strokes in terms of pathophysiology, risk factors, clinical presentation, and management [6,7]. This case highlights an important and well-documented yet frequently underappreciated risk factor for CVT-OCPs. The association between OCP use and venous thromboembolism, including deep vein thrombosis, pulmonary embolism, and CVT, has been extensively studied, with estrogen-containing contraceptives playing a pivotal role in the pathogenesis of thrombosis.

OCPs contribute to a hypercoagulable state through multiple mechanisms, including increased levels of coagulation factors (fibrinogen, factor VII, factor VIII, and factor X), reduced levels of anticoagulant proteins (protein S and antithrombin III), and impaired fibrinolysis. The risk of thrombosis is particularly heightened in individuals with additional prothrombotic conditions, such as inherited thrombophilias, prolonged immobilization, or dehydration [5,7]. However, in our patient, an extensive thrombophilia workup was negative, reinforcing the role of exogenous estrogen as the primary etiological factor. This finding underscores the need for careful risk stratification before prescribing OCPs, particularly in women with additional thrombotic risk factors.

The clinical presentation of CVT is highly variable, ranging from isolated headaches to focal neurological deficits, seizures, and even coma in severe cases. In this patient, the initial presentation of a progressive headache, followed by right-sided hemiparesis and dysarthria, reflects the common but often non-specific nature of CVT symptoms [2-5]. Headache is the most frequently reported symptom, occurring in up to 90% of cases, often due to increased intracranial pressure from impaired venous drainage [1,3]. However, because headache alone is a common and non-specific symptom, diagnosis is frequently delayed or missed. In this case, the presence of neurological deficits prompted further neuroimaging, leading to the timely identification of the underlying pathology.

Neuroimaging remains the cornerstone of CVT diagnosis. Non-contrast CT of the brain, although often the first imaging modality performed in suspected stroke cases, has low sensitivity for CVT, detecting direct signs (hyperdense cortical veins or sinus thrombosis) in only a subset of patients [4-7]. In our case, subtle hyperdensity along the superior sagittal sinus on CT raised suspicion, which was subsequently confirmed by CTV and MRV. MRV remains the gold standard for visualizing cerebral venous structures and evaluating sinus patency. The presence of venous infarction, as seen in our patient, indicates parenchymal injury due to venous congestion and carries a higher risk of neurological deficits.

The cornerstone of CVT management is early anticoagulation, even in the presence of venous infarcts. LMWH was initiated promptly in this case, followed by a transition to oral warfarin with INR monitoring [2,3]. Despite historical concerns about anticoagulation in the setting of hemorrhagic infarcts, current guidelines support its use, as the primary pathology in CVT is thrombotic occlusion rather than hemorrhage. Emerging data suggest that direct oral anticoagulants (DOACs) may be a viable alternative to warfarin, offering a more convenient and potentially safer profile, although further randomized trials are needed to establish their role in CVT management [5-7].

An important aspect of CVT care is long-term follow-up, including assessment of recanalization and consideration of anticoagulation duration. In this patient, repeat MRV at three months demonstrated partial recanalization, with complete resolution of symptoms at six months. Current guidelines recommend anticoagulation for at least 3-6 months in provoked CVT, with longer durations considered in cases with persistent risk factors or recurrent thrombosis. Given the clear association with OCPs, our patient was strongly advised to discontinue estrogen-containing contraceptives permanently and counseled on alternative contraception options [3-7].

This case underscores several critical lessons in CVT management. First, a high index of suspicion is required for early diagnosis, particularly in young female patients with progressive headaches and neurological deficits. Second, timely neuroimaging with MRV or CTV is essential for accurate identification. Third, immediate anticoagulation is key to preventing disease progression, even in the presence of infarction. Lastly, long-term monitoring, including follow-up imaging and risk factor modification, is crucial for optimizing patient outcomes and preventing recurrence.

Despite advances in CVT diagnosis and management, several knowledge gaps remain. The optimal duration of anticoagulation, the role of DOACs as first-line therapy, and the need for routine follow-up imaging are areas requiring further research. Additionally, greater awareness among primary care providers and emergency physicians is needed to reduce diagnostic delays and improve patient outcomes. Future studies should also focus on personalized risk assessment strategies for women using hormonal contraceptives to better balance the benefits and risks of OCPs.

## Conclusions

CVT is a rare but serious condition that requires a high index of suspicion, particularly in young women using OCPs. This report highlights the critical role of estrogen-containing contraceptives in promoting a prothrombotic state, reinforcing the need for individualized risk assessment before OCP initiation. Early recognition, prompt neuroimaging with MRV, and immediate anticoagulation are paramount in improving patient outcomes. Long-term follow-up, including assessment of recanalization and discontinuation of hormonal therapy, is essential to prevent recurrence. As research continues to refine treatment strategies, heightened awareness and early intervention remain key in mitigating the neurological consequences of OCP-associated CVT.
